# Bayesian Dynamical Systems Modelling in the Social Sciences

**DOI:** 10.1371/journal.pone.0086468

**Published:** 2014-01-20

**Authors:** Shyam Ranganathan, Viktoria Spaiser, Richard P. Mann, David J. T. Sumpter

**Affiliations:** 1 Department of Mathematics, Uppsala University, Uppsala, Sweden; 2 Institute for Futures Studies, Stockholm, Sweden; University of East Piedmont, Italy

## Abstract

Data arising from social systems is often highly complex, involving non-linear relationships between the macro-level variables that characterize these systems. We present a method for analyzing this type of longitudinal or panel data using differential equations. We identify the best non-linear functions that capture interactions between variables, employing Bayes factor to decide how many interaction terms should be included in the model. This method punishes overly complicated models and identifies models with the most explanatory power. We illustrate our approach on the classic example of relating democracy and economic growth, identifying non-linear relationships between these two variables. We show how multiple variables and variable lags can be accounted for and provide a toolbox in R to implement our approach.

## Introduction

Social science usually aims to explain macro-level phenomena, such as stratification, segregation, democratisation, economic development and changes in values. From the vast number of examples studied across sociology, politics and economics, a few examples include: Does inequality decrease or increase ethnic segregation [1]?; Do economic growth and changing cultural values of a society promote democracy [2], [3]? What are the causes of economic growth [4]? How does the structure of a social network affect opinion dynamics [5]? In these wide ranging questions, the macro-level variables can concern a variety of scales, from schools and neighbourhoods, up to companies and countries. The questions about them are similar: from observed macro-level patterns, can we work out the relationships that characterize these patterns [6].

While it is widely recognized that understanding at the micro-level is the key to causal mechanisms in sociology [7]–[9], it is possible to gain some understanding of social systems through macro-level patterns alone. One example of macro-level inference is fitting sigmoidal and saturating curves to describe diffusion of innovations [10], [11]. In this case, the type of growth curve is hypothesized to differ depending on whether innovation is driven by endogenous or exogenous factors at the micro-level. Specifically, by fitting the growth curve
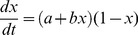
(1)to the proportion of individuals adopting a particular activity 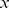 over time 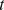, we can infer the relative importance of exogenous and endogenous (social) factors from the relative weights of the parameters 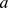 and 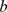, respectively. While having certain known limitations [12], [13], this approach has been usefully applied in the context of, for example, medical innovations and radio airplay.

Only a very small subset of social systems are characterized by sigmoidal growth curves. However, non-linear interactions between variables in social systems are common, and using differential equations to give an initial insight into macro-level relationships has a great deal of potential [14]–[16]. Econometrics provides a starting point for such an analysis. For example, in growth econometrics cross-country data is used to find which factors promote economic growth [17]–[19]. However, growth econometric analyses usually focus on the rate of change of one variable as a function of many potential factors, rather than dynamic interactions between variables. It is precisely these dynamic feedbacks which are of most interest in the social sciences and where reliable statistical approaches are required [4]. In recent years, detailed data describing long term changes in social systems has become widely available. For example, a variety of indicators now measure changes in the economics [20], [21], social development [21], political systems [22]–[24] and cultural values [25] of different countries and local regions. Identifying relationships between these macro-level indicators poses new challenges, but also opens up new opportunities. These challenges are not unique to between country comparisons, but arise in everything from social movements, workplaces, and neighbourhoods, down to modeling individual panel data on emotion dynamics [26], [27].

The approach we take here is inspired by machine-learning and algorithmic modeling [28], [29], in that we use the available data to inform model selection from a pool of feasible models rather than testing specific models against data. We use Bayesian model selection as a measure of the reliability and robustness of the differential equation models, basing our selection on the Bayes factor of each model. Our aim is to identify potential relationships in macro-level data, that can be further investigated in terms of mechanisms at the micro-level. Identifying this link is achieved by fitting polynomial differential equations, because they describe how change in an indicator variable occurs as an explicit function of the state of other indicators. This stands as a contrast to other function-fitting frameworks used in machine-learning, such as artificial neural networks or Gaussian processes, where the relationship between the variables is more opaque. In the preface to their book, Rasmussen and Williams [30] argue that statistics is concerned with understanding data and relationships in terms of models, whereas machine-learning is more focused on making accurate predictions. Mackay [31] (chapter 45) also highlights the limitations of advanced machine-learning methods for identifying ‘features’ in the data. While our methodology is inspired by such algorithmic model-search methods of machine-leaning, our goals are aligned with those of statistics and, of course, sociology, to identify explicit and clearly understandable relationships in the data.

**Figure 1:**
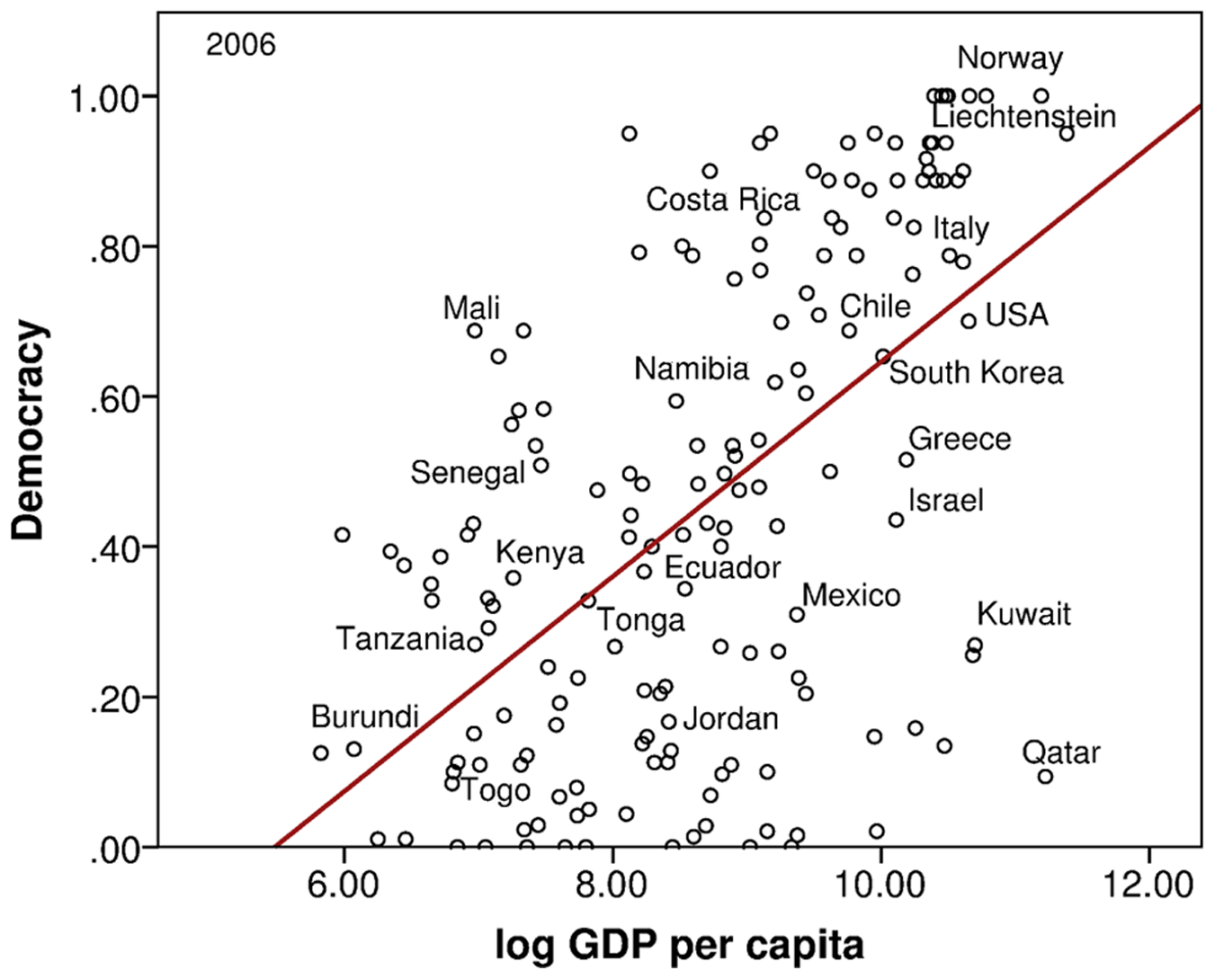
Country-based correlation scatter plot for log GDP per capita and Democracy for the year 2006. The Pearson correlation coefficient between the two variables is 0.571 (p<0.01).

We illustrate our approach on the classic problem of determining an interaction between GDP per capita and democracy (Fig. 1), which are known to correlate while the causal link and specific character of their relations remains an issue of debate [32]–[36]. The analysis has been performed on a set of 74 countries from 1981 to 2006. We implemented the method of Bayesian Dynamical Systems Modelling in an R package that is now openly available (Bayesian Dynamical System Model, *bdynsys*, to be found on CRAN (http://cran.r-project.org) and we used this package to do the analysis on the relation between GDP per capita and democracy. Our intention in this paper is not to speculate over why certain relationships exist, but rather to outline the methodology for identifying existing relationships.

## Methods

Our basic approach to understanding interactions between indicator variables is to model changes in one variable between times 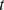 and 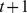 as a function of all included model variables at time 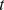. Suppose that we are studying a social system with 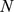 indicator variables 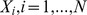. Let us assume that we have longitudinal or panel data for the 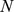 variables for 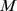 entities (such as countries, organisations, individuals etc.) over a length of time 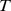. We denote the data as 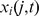 where 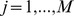 and 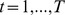. The changes in the variables over a time period are denoted as 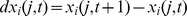. We write 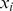 to denote the 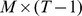 matrix (the values at time 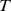 are not used in the modeling so we drop them from this matrix) whose elements are given by 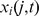. Similarly, 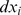 is the 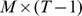 matrix with elements 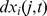. We now outline our approach for two variables and then move on to the general case.

### Systems with Two Variables

Our aim is to take these time series and fit an ordinary differential equations model to them. A system of differential equations with two variables can be represented as
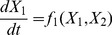
(2)

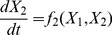
(3)for some appropriate functions 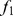 and 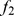. Note that since we have discrete data, we need to use difference equations instead of differential equations when performing the fitting. However, we express our model in terms of continuous time both for mathematical convenience and because the underlying social process is often continuous.

We can think of the time series of the indicator variables for each entity 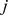 as corresponding to different trajectories obtained from the same system. We assume that any individual entity on reaching a certain state, represented by a unique set 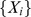, will experience the same effect, with distortions due to random, uncorrelated noise. The advantage of this approach is that we can use data arising from different initial conditions in different entities to build up an estimate of the functions 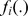. The potential disadvantage of this approach arises in cases where there are systematic differences between entities. This limitation should be borne in mind when we consider the results of the fitting.

In order to model as many non-linearities as possible, we take 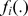 to be polynomial of sufficiently high degree. For computational purposes, we assume that the functions are polynomial in the indicator variables with each term being of degree 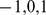 in the variables or a product of such terms. We also allow for terms that are quadratic and cubic in single variables. This keeps the number of evaluated models sufficiently small, while still allowing us to capture complex interactions. Higher order terms are important because they typically result in multi-stable states, which are characteristic of realistic social systems [16].

In our standard implementation of a two variable model, we study models of the form:
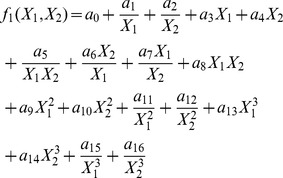



A model is defined by a subset of coefficients 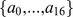 obtained by multiple regression of the change variable with the corresponding polynomial terms. The number of terms 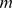 in the model is given by the number of non-zero coefficients. Thus there are 17 models with one term and 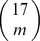, models with 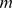 terms. The objective of the model selection algorithm is to choose among these models the one that best fits the data. We do this in two stages.

In the first stage, we aim to rapidly narrow our search by finding the maximum-likelihood model for each possible number of terms 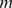. We fit the changes in the indicator variables using multiple linear regression over all 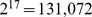 possible functions 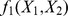. For all models we compute the log-likelihood value
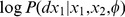
(4)where 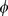 is the set of parameters that defines each specific model. Assuming that errors are due to additive Gaussian noise, finding the maximum log-likelihood is equivalent to finding the minimum of the sum of squared errors (SSE) scaled by the variance [29], i.e.,
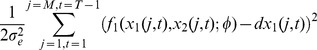
(5)For each number of terms 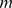 we determine the best fit model 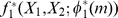, where the parameter set 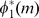 maximizes the log-likelihood over all models with 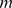 terms.

We assume that the noise variance 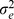 is equal to the data variance. If the noise variance is unknown, the posterior distribution will not be Gaussian and hence maximizing the likelihood might not be equivalent to minimizing the SSE. But we can use Monte Carlo methods to compute the log-likelihood value by integrating over all possible noise variances provided we know a prior distribution for 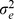. Note that the sum of squared errors can be used to calculate the coefficient of determination or the 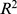 value as
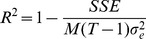



Instead of reporting 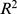 values for our models, we report the maximum log-likelihood values
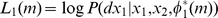



In the second stage of our model selection algorithm, we choose the best model among those obtained in the first stage based on their ‘robustness’. Clearly, 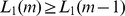 for all 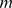, that is, the maximum log-likelihood value increases monotonically with number of terms, since each term allows an extra degree of freedom on curve fitting (*in extremis* the additional coefficient can be set zero to obtain the same likelihood). For a finite data set, this extra degree of freedom can fit artifactual patterns due to noise. As a result, reliance on 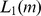 alone can lead to overfitting the data by selecting too many terms and thus accepting a model that accurately fits the existing data but that generalises poorly to unseen data and has little predictive power (see [31] Chapter 20).

To address this problem and evaluate the fit of these models, we adopt a Bayesian approach [31], [37]–[40]. We calculate the Bayesian marginal-likelihood [31], [41] or the *Bayes factor* 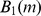 for the set of models which have the largest log-likelihood value among models with their respective number of terms. Note that ‘Bayes factor,’ which refers to a *ratio* of model likelihoods is used in Bayesian literature to compare pairs of models [37], [42]. We use the same term in this paper to refer to the Bayesian marginal likelihood as defined above, with the understanding that this measure would have the same function as the Bayes factor when comparing between more than two models.

The Bayes factor compensates for the increase in the dimensions of the model search space by integrating over all parameter values [31], i.e.,
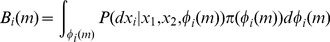
(6)where 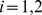. The Bayes factor is thus the likelihood averaged over the parameter space with a prior distribution defined by 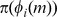. The prior distribution says how likely we thought a particular parameter value was before we started the fitting procedure [29]. We choose a non-informative prior distribution, such that, 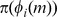 is uniform over a range of parameter values. This reflects our lack of knowledge about what parameter values might arise. In many social systems, domain experts may actually have prior knowledge and this can be directly incorporated into 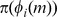. We do not do incorporate such information here, but envisage that it could be an important part of combining our data-driven methodology with existing knowledge.

In our implementation the range of values for 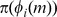 is chosen to include all feasible values but to be small enough for the integral to be computed using Monte Carlo methods. 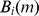 is computationally expensive to calculate. Therefore we use the two stage algorithm described above, since models of equal complexity (same number of terms in this context) can be more fairly evaluated in terms of their log-likelihood values.

We compute the Bayes factor for the models 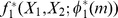 with different numbers of terms 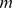 and obtain the function 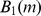. Using this and other background information, we can choose the best model. For example, in the absence of any prior information, the model with the highest Bayes factor is the optimum choice. However, there may be theoretical considerations for the particular system being studied that makes another model preferable even if it does not have the highest Bayes factor. In this case, we could, in theory, incorporate this information when choosing the prior distribution for the parameters. But where that is not straightforward, we use the Bayes factor plot to serve as a guide in comparing between the models while choosing the most efficient one for the problem being studied.

We repeat the same process to obtain the Bayes factor plots for the 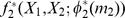 models, in order to calculate 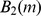.

In our implementation, and the application given below, we choose to use a uniform prior distribution for 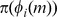. The non-informative prior is reasonable when we have no information about the parameter space except its likely range. If we know the parameter distribution is symmetric and the parameter is less likely to be a large value, then a normal distributed prior is a reasonable assumption. In this case, the Bayes factor can be evaluated directly and we do not need to use a Monte Carlo evaluation. Specifcally, suppose 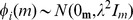. The integrand in equation 6 is then the product of normal distributions and the integral is itself a normally distributed random variable with mean 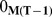 and covariance matrix given by 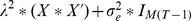 evaluated at the observed values of 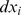 [42]. Here 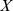 is the 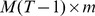 matrix of the observed values of 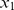 and 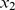 modified according to the terms chosen in the particular 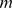-term model we are evaluating.

### Systems with More than Two Variables

Most social systems have many interacting variables and we can easily extend our methodology to systems with more variables. In this case, we need to get the best possible models 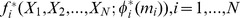 using 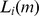 and 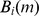 as described in the two variable case. The main constraint to this approach is that the number of possible models that need to be evaluated grows exponentially with number of variables. For models with three variables, with the polynomial functions that we look at, there are 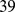 possible models with one term and 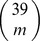 models with 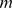 terms. Due to the computational complexity involved, we only search the model space for models with up to 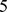 terms in this case.

One approach to solve this problem is to use a model pruning algorithm that will search only a fraction of the entire model space by looking only at models with higher terms that are extensions of the best models with fewer terms. For instance, suppose 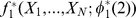 was the best two term model and is represented by the non-zero coefficients 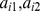. Then, when evaluating three term models we only look at models that have these terms and an additional term 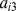. In this manner, we restrict the number of models that we need to evaluate to a very small number. Clearly this might not be optimal in all cases, so instead of extending only the best 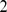 term model, a better strategy would be to evaluate all 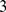 term models that are extensions of, say, the 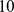 best 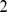 term models.

### Lag and Lead Times

In social systems, there is often a lag or lead effect in variables. A change in one variable at any instant 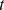 is often not a function of the level of another variable at 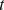 but of its level a few timesteps in the past. The approach as we describe above already captures autocorrelation, because we fit 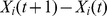 as a function of 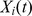, but it does not capture second order effects where, for example
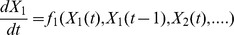



To handle this issue, we can extend our approach by including as a new variable the time-lagged variable of interest. For instance, in a two variable system, if there is evidence to suggest that 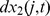 may be a function of both 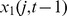 and 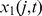, a simple implementation would be to look at the three variable system comprising 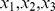, where 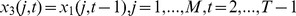. Now we can compute the best possible models 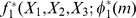 and 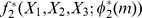 to understand the effects of lagged variables.

### Correlated Noise

In the methodology described above, best fit regressions on 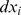 are performed assuming that the errors are uncorrelated to obtain the best possible models 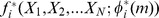 with 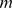 terms. But often the distortion or the noise in the socio-economic process occurs due to systematic reasons, for instance due to latent variables that affect all the model variables simultaneously, and hence is correlated across the variables. We have to account for the correlated noise in finding the best fit models and we handle this using the seemingly unrelated regressions approach [43].

Consider, for simplicity, the two variable system. The residual errors for the models 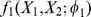 and 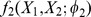 are given by 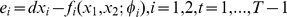 where the 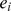 are 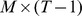 matrices. In performing least squares regression, we used the standard assumption that the errors are independent Gaussian processes. Thus, 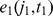 the error in the model for the first variable for entity 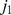 at time 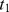 is independent of 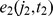 the error in the model for the second variable for entity 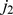 at time 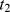 for any 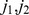, 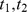.

In the case of correlated noise processes, 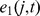 and 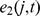 are correlated whereas the errors in different entities and at different times are still uncorrelated. Thus in this model, we have the covariance matrix defined by
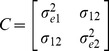
where the noise variances are 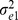 and 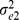, and the noise covariance is given by 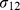. In the 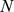 variable case this is a symmetric 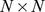 matrix with the elements defined similarly.

In the seemingly unrelated regressions approach, we obtain the regression coefficients 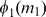 and 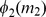 for the models 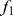 and 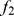 as before. To calculate the regression coefficients in the presence of correlated noise, we use these coefficients and compute the observed error covariance matrix as
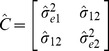
where the diagonal terms are computed as



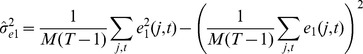
and similarly for 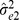. The off-diagonal elements are given by



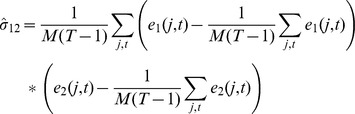



Using this estimated covariance matrix, we perform multiple regression using the standard methods for regressions in the presence of correlated noise [42] and re-estimate regression coefficients. Using the new coefficients we re-calculate the errors and the estimate of the noise covariance matrix as detailed above and iterate this process until the regression coefficients converge. This typically happens in a few steps, but if convergence is not achieved in a fixed number of steps a stopping criterion can be set.

If the underlying noise covariance matrix is almost diagonal, indicating that error terms are uncorrelated, the parameters estimated by the seemingly unrelated regressions approach will not differ significantly from the parameters obtained assuming uncorrelated errors.

## Results

We now investigate a frequently studied macro-level phenomenon, the relation between democracy and GDP per capita, using our proposed methodology [32]–[36]. We used data for GDP per capita that is provided by the World Bank (http://data.worldbank.org), and a democracy index [44], based on the Freedom House political rights and civil liberties scores [22], [23], weighted by the countries’ human-rights-performance, measured by two indices from the Cingranelli/Richards Human Rights data project [24], [25]. We modeled the interaction of democracy and GDP per capita for the years 1981–2006 for 74 countries.

**Figure 2:**
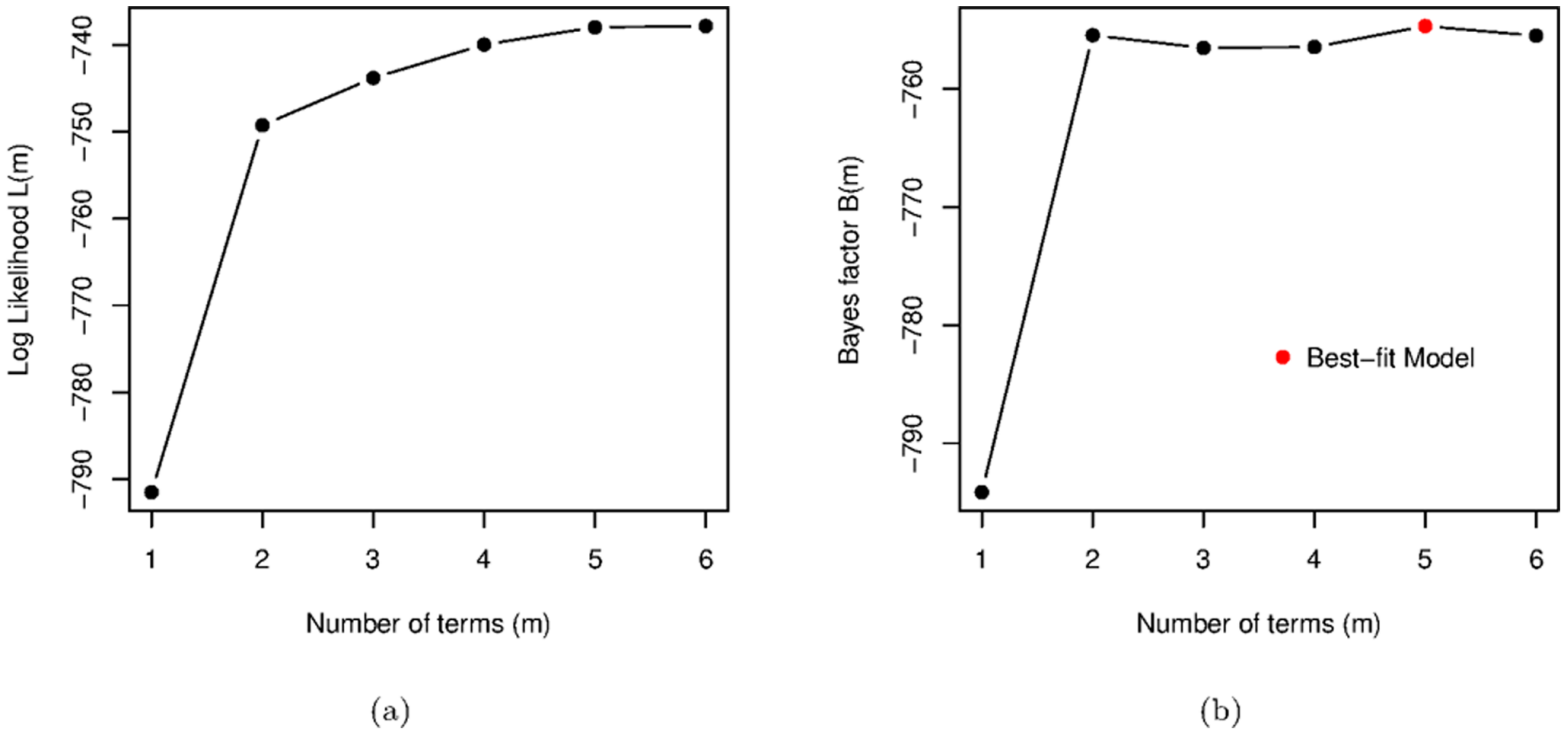
Change in Democracy models. (a) The Log Likelihoods and (b) Bayes Factors for the same models.

We start our analysis with two variable models, in which changes in democracy (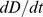) are a function of democracy itself (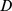) and log GDP per capita (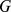). Figure 2 shows that the maximum log-likelihood 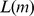 for this model strictly increases with the number of terms 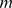. The Bayes factor 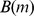 grows significantly when we add a second term, decreases slightly for 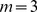 and 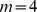 terms, before increasing again for 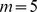 terms and decreasing finally when a sixth term is added.

The best two term model for changes in democracy is
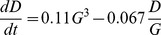
(7)


In words, this model tells us that democracy grows once GDP per capita has reached a certain threshold, with this threshold being determined by democracy itself. Specifically, democracy grows when 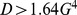.

The best five term model, and the best overall model is
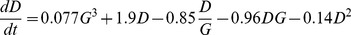
(8)


As such complex models are usually more difficult to interpret in words, we might prefer the second best model with just two terms whose Bayes factor is only slightly smaller. Accepting simpler models makes interpretation of the interaction of GDP per capita and democracy more straightforward. On the other hand, the Bayes factor has already taken model complexity in to account, and we should look carefully at what the more complex model tells us.

**Figure 3:**
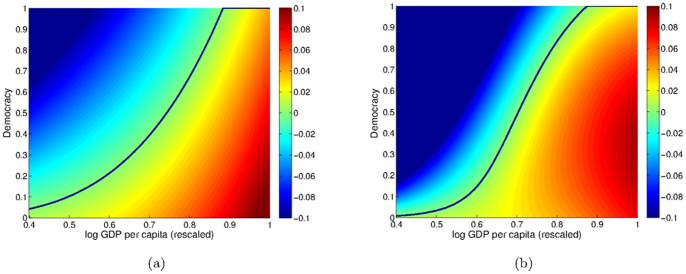
A heatmap of change in democracy models. For (a) the two term model given by equation 7 (b) the five term model given by equation 8. The colour scale gives the rate of change of democracy (i.e. 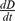) as a function of 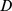 and 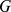. The black line is the solution 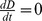.

We can investigate the difference between the two and five term models by visualizing the functional form of the two 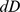 models (see Fig. 3). In this figure, the blue colours represent where democracy decreases and the yellow/red colours where democracy increases. The threshold for transition from positive to negative growth is approximately the same in both models (black line in Fig. 3), although the five term model has a point of inflection. A more important difference is that, countries with high GDP but low democracy (bottom right-hand corner of Fig. 3), experience slower growth in democracy in the five term model than in the two term model. The additional flexibility of the five term model thus captures the slow growth in democracy of rich, but undemocratic countries.

**Figure 4:**
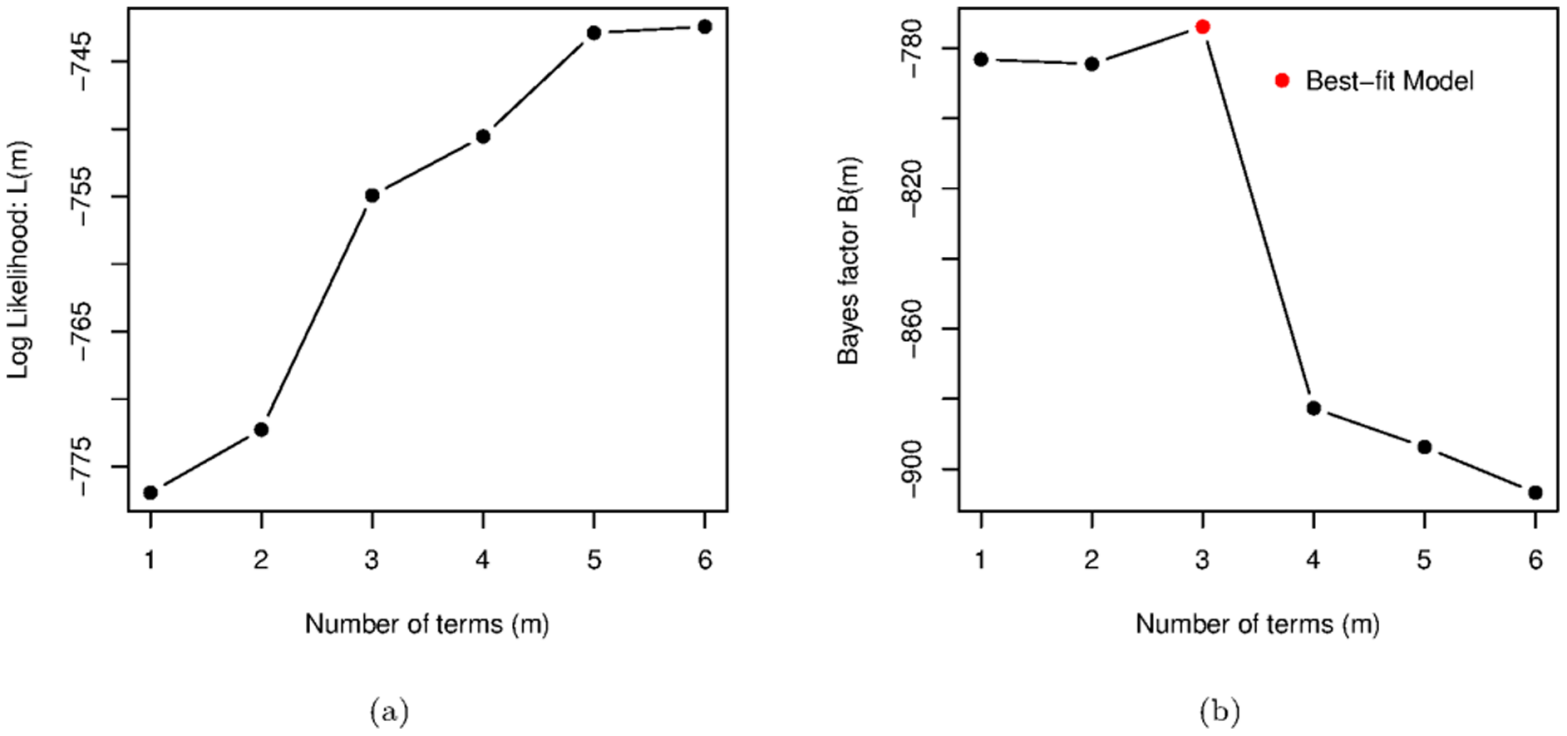
Changes in GDP per capita models. (a) The Log Likelihoods and (b) Bayes Factors for the same models.

Now that we understand better what impact GDP per capita has on democracy, we turn our attention to changes in GDP per capita, 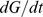. The best model (Fig. 4) for 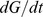 as a function of GDP itself and democracy is,
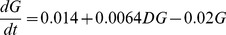
(9)


The model shows that GDP is primarily growing at a constant rate, but is additionally positively affected by democracy interacting with GDP. Moreover, the growth is self-limiting at high levels of GDP.

**Figure 5:**
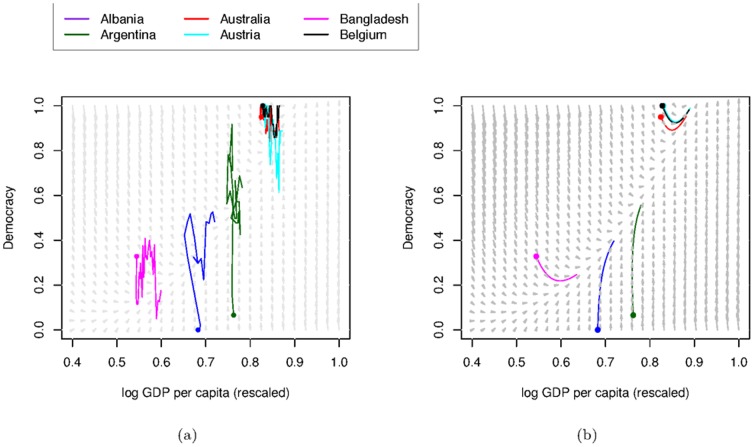
Phase portraits with trajectories. From (a) country by country data and (b) model predictions made by integrating equations 7 and 9, with coloured circles representing the initial conditions. The arrows in the phase portrait indicate the direction and magnitude of the changes 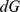 and 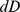 as a function of the two variables 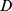 and 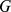 themselves. Specifically the arrows are the vector 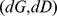 given by equations 7 and 9. These arrows are the same in both sub-figures.

With the interactions between democracy and GDP per capita identified, they can be displayed visually using a phase portrait We provide phase portraits with example trajectories both from the data (Fig. 5a) and the model-predictions made by numerically integrating equations 7 and 9 (Fig. 5b). The model predicts quite well the general trajectories of the countries. For example, increases in democracy for Albania and Argentina and the initial decrease then increase in democracy in Bangladesh. The model is deterministic and therefore does not capture fluctuations seen in the data, but the overall pattern seen for specific individual countries is reproduced.

We now implement the various extensions presented in the methods section. We start by testing for correlated noise in the models. Computing the error correlations for equations 7 and 9 gives an error correlation of 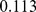. This correlation is not big, but possibly not negligible either. Accounting for error correlation and re-estimating parameters results in changes of the coefficients:
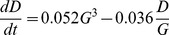
(10)

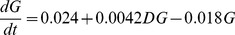
(11)


The re-estimated coefficients are only slightly different than those originally determined and do not make a significant difference to the phase portrait. Generally, it appears that the effects of both democracy on GDP per capita and of GDP on democracy are slightly overestimated using the original method.

A natural extension to three variables in this case is the inclusion of a lag. In particular, we might expect earlier levels of GDP to influence future changes in democracy. We therefore look at changes in democracy 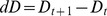 as a function of itself (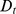), GDP per capita (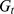) and lagged GDP per capita (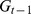). We now use the additional subscript to make clear the lags involved. Using 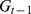 as an additional variable results in the following best model:
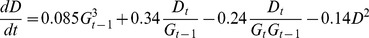
(12)


**Figure 6:**
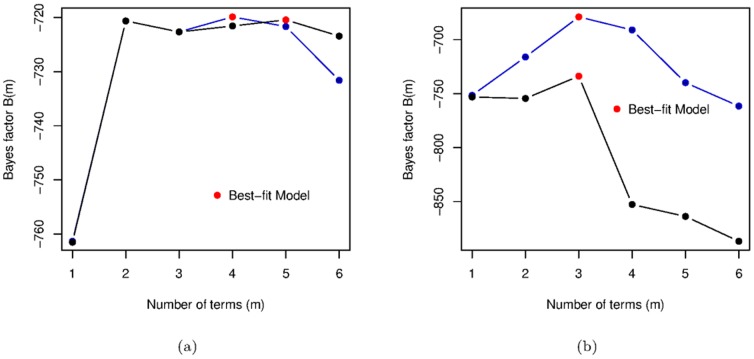
Choosing the best models. (a) Bayes factors for the two-variable 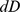 models (black) and for the three-variable 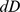 models (violet). The two red marked models refer to equation 8 and equation 12. (b) Bayes factors for the two-variable 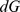 models (black) and for the three-variable 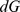 models (violet). The two red marked models refer to equation 9 and equation 13. In both cases, the Bayes factor for the two and three variable models are calculated over the same subset of data, which is (necessarily) one year shorter than that used in Fig. 2 and Fig. 4.

The Bayes factor for both three variable lagged and two variable non-lagged models are shown in Fig. 6. While this model does have a better Bayes factor than both the two and five term non-lagged models, the difference is marginal. Moreover, equation 12 resembles the best five-term model for changes in democracy in equation 8. 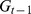 has a positive non-linear effect on democracy, and democratic growth remains self-limiting. These observations lead us to conclude that the additional complexity added by the lagged term outweigh the marginal improvement in goodness of fit.

The best model (see Fig. 6) for changes in GDP per capita when fitting a three term model with 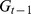 is
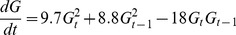
(13)


**Figure 7:**
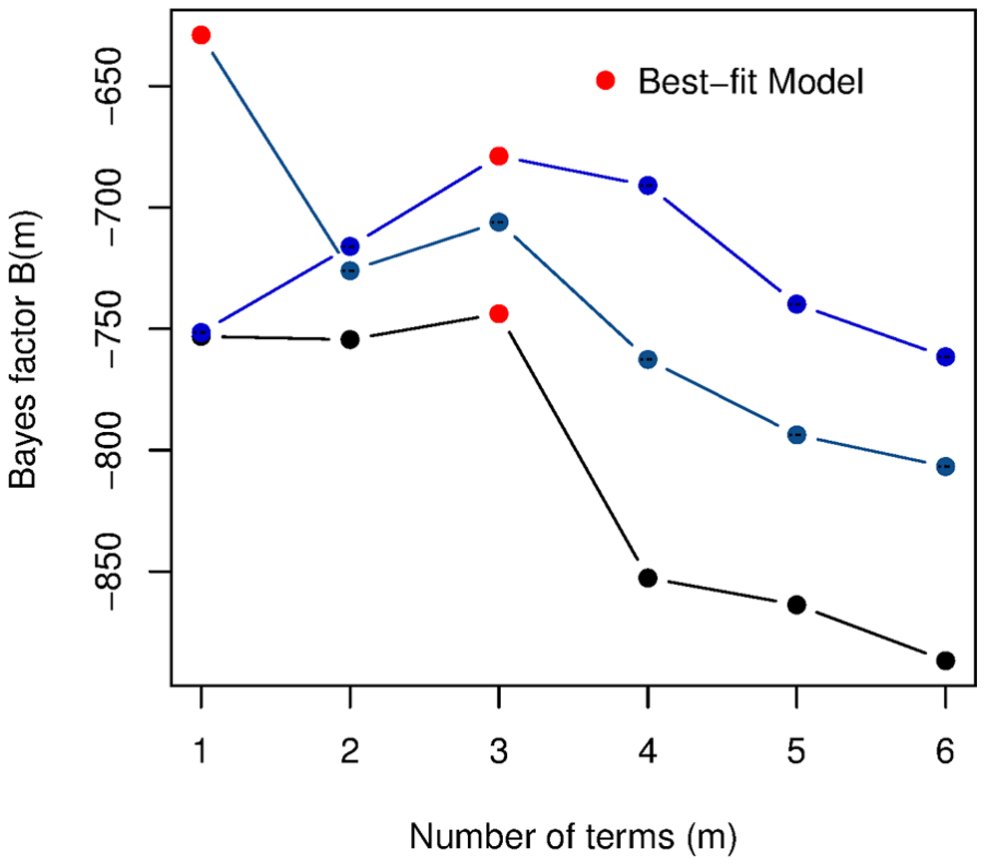
Bayes factor plots. For the two-variable 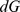 models (black), the three-variable 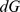 models with lag GDP per capita (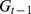) (violet) and the three-variable 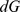 models with 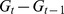 as the third indicator variable (blue).

Here, democracy is no longer a predictor for GDP per capita, which is now solely predicted by itself at different points in time. This is now a significantly different model than the one given by equation 9. Moreover, the model in equation 13 fits the data significantly better than the model given by equation 9 (see Fig. 6). It is possible that in the two variable models democracy, to an extent, played the role of the lag variable, rather than acting as a proper predictor of change in GDP. We then further note that factoring equation 13, we get
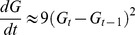
(14)suggesting that the growth in 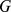 between times 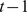 and 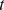 is the best predictor of changes between times 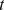 and 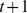. This possibility is confirmed if we use 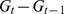 as the third indicator variable. Now, fitting 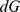 as a function of 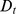,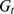 and 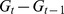 we find that the best model for changes in GDP per capita has only a single term,



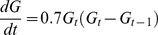
(15)This models turns out to be the overall best model for changes in GDP per capita (Fig. 7).

For completeness, we also checked whether changes in democracy improve with 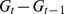 as the third indicator. In this case, the best model is again equation 8, and the best two term model is equation 7, indicating that lags in GDP are not important in modeling changes in democracy.

## Discussion

The approach to studying social systems we present here emphasizes exploratory model fitting. Exploratory approaches help to identify new and unexpected patterns and explanations [46]–[48]. Such an approach is not completely atheoretical, since we need to think about which variables to investigate. However, instead of defining how the variables should interact and then testing this pre-defined relation in the data, we allow the data to inform us about the mathematical linear or non-linear relationships between the indicator variables. This does not imply that our approach is only applicable in cases where there is no existing theory. Indeed, if a theory can be expressed in terms of relations in a differential equation then it can also be compared, via Bayes factor, to other alternative models.

Our methodology can be applied to any social system which has reasonable amounts of longitudinal or panel data, that is data with repeated measurement over time for a number of independent entities. On the macro-level the method can be used to study cross-national development dynamics, for instance, the relationship between a country’s gross domestic product, child mortality and education levels. If regional or city district data is available it is possible to use the method to study for instance neighbourhood segregation processes. On a meso-level the researched entities could be organisations, companies or schools, to study, for instance, dynamic female employment patterns of companies.

In studying social systems there is seldom one single unique best fit model that fully explains the data. As we saw when comparing the Bayes factor of equations 7, 8 and 12, several models provided a robust fit to data. The advantage of using Bayes factor is that complicated models are automatically punished. Including more terms does not necessarily improve fit, since all of the extra parameters are included in the stochastic integration in equation 6. For example, the complex non-linear interaction (given by equation 8 and visualized in in Fig. 3) whereby rich, undemocratic countries showed slower democratic growth than some poorer and more democratic countries gives only a slightly more robust fit to data than the simpler two-term interaction (given by equation 7 and visualized in Fig. 3). At this stage, we should accept both as plausible models.

We do not attempt here to give a political or sociological interpretion of the best fit models. However, the dynamic nature of the fitted models provides a starting point for thinking about causal mechanisms [7], [8]. For example, we see that the best model for explaining change in democracy as a function of economic development involves a threshold. When GDP is sufficiently high, democracy typically increases. This observation certainly provides more understanding than the correlation presented in figure 1, but it does not fully open up the black box relationship between the two indicators. In order to open up this black box, we need to investigate both the role of other indicator variables in linking democracy and economic growth, as well as proposing possible micro-level mechanisms which can explain the transitions to democracy.

The suggestion that the terms in the fitted models could relate to plausible causal mechanisms is one argument for adopting an approach based on differential equations. If our aim was only to predict future changes in indicator values then any one of a range of statistical or machine learning techniques, such as Gaussian processes or neural networks, could be employed in model fitting. We could further employ Bayesian model averaging in which we weight models according to their Bayes factor [19]. While these techniques are undoubtedly of use in making predictions, differential equations lend themselves more readily to interpretation. The use of differential equations has been the strength of earlier work using logistic growth to model the diffusion of innovations [10]. Fitting models has informed debate over micro-level mechanisms [12], [13]. The methods outlined here provide a rigorous extension of the differential equation approach to a general set of interactions between multiple indicators.
